# Varying behavioral differences and correlates of HPV infection among young adolescents in Benue state, Nigeria

**DOI:** 10.1186/s12889-024-19068-7

**Published:** 2024-06-07

**Authors:** Evelyn Erdoo Orya, Kayode Khalid Bello, Sidney Sampson, Esther Awazzi Envuladu, Hilary I. Okagbue

**Affiliations:** 1 Sydani Group, Abuja, Nigeria; 2 Federal Medical Centre, Keffi, Nasarawa State Nigeria; 3 Jos University Teaching Hospital, Katon Rikkos, Plateau State Nigeria; 4 Sydani Institute for Research and Innovation, Sydani Group, Abuja, Nigeria

**Keywords:** Benue State, HPV, Nigeria, Public health, Sexual behavior, STI, Vaccination

## Abstract

**Background:**

Human Papillomavirus (HPV) infection is a significant public health concern globally, especially in low- and middle-income countries. In Africa, including Nigeria, HPV prevalence is high, contributing to a substantial burden of cervical cancer. Despite challenges, massive HPV vaccination campaigns in Africa show promise for preventing cervical cancer cases. In Benue State, Nigeria, limited research exists on several aspects of HPV knowledge and attitudes towards HPV among secondary school students. This study aims to bridge this gap by assessing HPV knowledge, prevention practices, willingness to uptake HPV vaccination, and associated attitudes and behaviors among secondary school students in the state.

**Methods:**

The cross-sectional study was conducted among adolescents aged 10–19 years in six secondary schools in three local government areas (LGAs) in Benue State, Nigeria. Two-stage sampling was used to select the LGAs and schools, with a final sample size of 591 students. The selected schools represent both junior and senior secondary school levels and span across the three senatorial districts of the state. Data were collected using a self-administered questionnaire covering sociodemographic characteristics, HPV knowledge, prevention practices, and willingness for HPV vaccination. Statistical analyses included univariate analyses and tests of association, with significance set at *p* < 0.05 or 0.001 depending on the level of the stringency of the evidence required. Data were analyzed using SPSS version 25.

**Results:**

Only 35.4% of the participants were males, and 86.8% were between the ages of 10 and 16. Only 24.7% acknowledged that HPV can be transmitted during sexual intercourse, and 36.2% recognized that HPV can be transmitted via skin-to-skin contact. 48.1% noted that HPV can cause cervical cancer. Half (50.9%) acknowledged that early sexual debut increases the risk of acquiring HPV, while only 28.1% recognized vaccination as a preventive strategy against HPV infection. Only 35% correctly stated the best time for the HPV vaccine. In assessing the practice of HPV prevention, 14.9% are in a sexual relationship and 10.3% admitted to not using condoms during sexual intercourse. Also, 11.8% have had STIs, and 27.2% have previously undergone HIV screening. Various bivariate analyses showed some varying behavioral differences and correlates of HPV infection among young adolescents in Benue State, Nigeria.

**Conclusions:**

This study provides valuable insights into HPV knowledge, prevention practices, and willingness to uptake HPV vaccination among secondary school students in Benue State, Nigeria. The significance of the differences and correlates was discussed using themes. The research has unpacked complex relationships that could have public health implications for researchers and policymakers. Moreover, ten actionable policy recommendations were prescribed. Several interventions and areas for further study were proposed.

## Background

Human Papillomavirus (HPV) infection is a significant global public health concern, particularly in low- and middle-income countries with weak healthcare systems [1]. HPV is one of the most prevalent sexually transmitted infections (STIs), with a lifetime exposure occurring in more than 50% of sexually active males and females [2]. HPV can lead to various cancers, including cervical, anal, penile, throat, and mouth cancers [3]. In Africa, the incidence of new HPV infections is high, contributing to the region’s burden of cervical cancer, which accounts for over 90% of cervical cancer cases globally [4]. HPV prevalence is among the highest in sub-Saharan Africa (SSA), at an average of 24% [5]. The current drive for massive vaccinations against HPV across Africa has the potential to prevent nearly most invasive cervical cancer cases. Various successes have been reported in massive HPV vaccinations despite the stubborn barriers that for years limited HPV coverage in Africa [6]. School-based health HPV interventions in Africa have been essential for reaching adolescents during their early adolescence, when they are particularly vulnerable to sexual health risks [7]. These interventions provide an ideal platform for delivering HPV peer education, which can significantly enhance adolescents’ knowledge about HPV, increase their use of testing services, and improve their acceptance of screening and vaccination [8]. Previous school-based interventions in Nigeria have largely concentrated on raising awareness of HPV and its vaccine among mostly female adolescents [9–12]. School-based HPV interventions in Nigeria are currently being deployed in HPV mass vaccinations as Nigeria targets to vaccinate 8 million girls [13]. However, there is insufficient evidence on the impact of these interventions, particularly regarding HPV vaccination [14], although several barriers and facilitators of the use of school-based HPV vaccination have been synthesized [15, 16].

In Nigeria, a lot of literature abounds on HPV research on different aspects, such as the high prevalence of HPV among women [17], massive vaccination [18], molecular epidemiology [19], barriers and facilitators of HPV vaccine uptake [20], HPV awareness [21], and others. However, only two studies have been reported for Benue State, Nigeria, which is the study area of this research. In the first paper, although a cross-sectional study was carried out to determine the prevalence and predictors of knowledge of HPV and HPV vaccine in different states of Nigeria, only 530 (20.9%) respondents (senior secondary (high) school students) are from Benue, and the sample selection was concentrated in only one senatorial district of the state [22]. In the second paper, which was aimed at assessing the knowledge, attitudes, and practices of HPV infection, cervical cancer, and HPV vaccination in Benue State, the respondents were pregnant women [23]. Hence, there is a research gap in the study area. This paper bridges the gap by extending the scope to the secondary schools in the three senatorial districts of the state and from students from junior secondary schools that have not been previously reported for Benue State.

This study aims to assess the knowledge of HPV infection and practice of primary prevention, the willingness of HPV uptake, and attitudinal differences and correlates among randomly sampled secondary school students across the three senatorial districts of Benue State. This study is unique in different ways. First, this is one of the few studies that included males in the sample. Second, this is the first study (to the best of the authors’ knowledge) to link HPV to other attitudes and behaviors such as age of sexual debut, substance abuse, condom use, alcohol and cigarette use, frequency of sexual intercourse, and human immunodeficiency virus (HIV) in Nigeria.

## Methods

### Study population and settings

The study population consisted of adolescents aged 10–19 years in selected secondary schools in three local government areas (LGAs) (Makurdi, Otukpo, and Ushongo) in Benue State. The state is located at latitude 7° 19’ 60.00” N and longitude 8° 44’ 59.99” E. The study was conducted in six selected secondary schools across the three LGAs of Benue State. These schools comprised two major levels: junior secondary school (JSS) and senior secondary school (SSS). The three LGAs represent the three senatorial zones of the state. This selection was made to ensure inclusion and to ensure that the samples are a true representative of the population. The study was carried out in 2022, a year before the roll out of HPV vaccines in Nigeria.

### Study design

The study design was a school-based cross-sectional study using a quantitative data collection approach. The samples consisted of school adolescents from secondary schools. This study follows a similar study design presented in [24].

### Sampling procedure

Two-stage sampling was utilized to select the three out of twenty-three LGAs in Benue State and the six schools. In the first stage, three LGAs were chosen from 23 LGAs from the three senatorial zones of the state using two-stage sampling to ensure representativeness. In the second stage, within each of the three selected LGAs, two schools were chosen, resulting in a total of six (6) schools. Within these schools, students were recruited using simple random sampling, which gives every student an equal chance of being selected, ensuring fair representation. The Cochran method, suitable for large populations, initially suggested a sample size of 384 respondents to achieve reliable findings. Anticipating that not everyone would respond or provide complete data, the researchers initially drew a larger sample size of 734 students. Out of the 734 students, 120 reported that they had not heard of HPV (Human Papillomavirus), and since awareness of HPV was necessary for the study, these students were excluded, reducing the sample size to 614. After conducting the survey, an additional 23 students either did not respond or provided only partial responses, resulting in their exclusion from the final analysis. Consequently, the final sample size used for the study was 591 students, providing the complete and relevant data needed for the research.

### Data collection procedures

A self-administered semi-structured questionnaire adapted from the Vaccination and HPV Knowledge (THinK) survey and the HPV Knowledge survey (New Zealand) was utilized to collect information from students [25, 26]. The questionnaire comprised five sections: sociodemographic characteristics, knowledge of HPV infection, knowledge of HPV primary prevention, practice of HPV prevention, and willingness for HPV uptake. Prior to the main study, the questionnaire was pretested on 10% of the sample population in Karu LGA, Nasarawa State which is distant from the study LGAs in Benue state. Trained interviewers administered the questionnaires to the students.

### Statistical analysis

Data were entered into the Microsoft Excel spreadsheet and cleaned. After cleaning, the data were exported into IBM Statistical Product and Service Solution (SPSS) version 25. Univariate analyses were conducted on the socio-demographic characteristics of students and were presented in frequencies and proportions. Chi-square tests of independence, Mann-Whitney tests, and Kruskal-Wallis tests were used for the test of association and median differences, respectively. A *p*-value less than 0.05 or 0.001 was considered significant depending on the level of the stringency of the evidence required.

## Results

### Univariate analysis

Table 1 presents the key sociodemographic characteristics of the 591 students. The sample consists of more females than males, with the majority identifying as Christians (97.6%), having parents with tertiary education (48.7%), and belonging to the Tiv tribe (53.0%). A significant portion, comprising 86.8%, falls within the age range of 10 to 16 years, while 67.8% have parents who are either civil servants or farmers. The distribution between Junior Secondary School (JSS) and Senior Secondary School (SSS) students is nearly equal in numbers.

**Table 1:** Basic Sociodemographic Characteristics of the respondents (*n* = 591)

Variable	Frequency	%
**Age group (Years)**		
10–13	239	40.4
14–16	274	46.4
17–19	78	13.2
**Sex**		
Male	209	35.4
Female	382	64.6
**Religion**		
Christianity	577	97.6
Islam	14	2.4
**Parent’s Occupation**		
Civil servants	209	35.3
Traders/Businessperson	21	3.6
Unemployed	49	8.3
Farmers	192	32.5
Others^+^	120	20.3
**Parent’s educational status**		
None	63	10.7
Primary	52	8.8
Secondary	188	31.8
Tertiary	288	48.7
**Tribe**		
Tiv	313	53.0
Idoma	211	35.7
Igede	35	5.9
^+^Others	32	5.4
**Class**		
Junior Secondary School	305	51.6
Senior Secondary School	286	48.4

Assessment of knowledge of HPV infection, knowledge of HPV primary prevention, prevention practices, and willingness to uptake HPV vaccination was conducted through the questionnaire, which aimed to assess understanding and behaviors. The four major factors were evaluated using frequencies and percentages.

Table 2 presents the findings regarding the knowledge of HPV infection. A notable discovery is that only 24.7% of respondents acknowledged that HPV can be transmitted during sexual intercourse, and merely 16.5% believed that HPV does not require any treatment. None of the variables recorded a 50% accurate understanding of HPV infection. Additionally, a significant finding is that only 36.2% of respondents recognized that HPV can be transmitted via skin-to-skin contact (non-sexual transmission). Nearly half (48.1%) of the respondents noted that HPV can cause cervical cancer.

**Table 2:** Knowledge of HPV infection

Code	Variable	Yes (%)	Don’t know (%)	No (%)
B1	HPV infection is an STI	242 (40.9)	64 (10.8)	285 (48.3)
B2	HPV can cause genital warts	278 (47.1)	171(28.9)	142 (24.0)
B3	HPV can cause cervical cancer	284 (48.1)	188 (31.8)	119 (20.1)
B4	HPV can cause penile cancer	271 (45.9)	178 (30.1)	142 (24.0)
B5	HPV can cause oropharyngeal cancer	209 (35.4)	159 (26.9)	223 (37.7)
B6	HPV can be transmitted via skin-to-skin contact	214 (36.2)	116 (19.6)	261 (44.2)
B7	HPV can be transmitted during sexual intercourse	146 (24.7)	172 (29.1)	273 (46.2)
B8	HPV infection can cause infertility	233 (39.4)	54 (9.1)	304 (51.5)
B9	HPV usually doesn’t need any treatment	98 (16.5)	258 (43.7)	235 (39.8)

Table 3 presents the findings regarding the knowledge of HPV primary prevention. Among the respondents, 50.9% acknowledged that early sexual debut increases the risk of acquiring HPV, while 54.5% correctly stated that abstinence from sex can prevent HPV infection. However, it is concerning that only 28.1% recognized vaccination as a preventive strategy against HPV infection. Furthermore, 44.7% believed that the HPV vaccine protects against all sexually transmitted infections (STIs). When assessing their knowledge of the optimal timing for receiving the HPV vaccine, 35% responded that it should be before sexual intercourse, 7.8% responded after sexual intercourse, 20.8% indicated it should be administered when someone is already infected with HPV, and 36.4% admitted to having no knowledge of the recommended timing.

**Table 3:** Knowledge of HPV primary prevention

Code	Variable	Yes (%)	Don’t know (%)	No (%)
C1	Sex at an early age increases the risk of getting HPV	301 (50.9)	56 (9.5)	234 (39.6)
C2	Abstinence from sex can prevent infection with HPV	322 (54.5)	64 (10.8)	205 (34.7)
C3	A person could have HPV for many years without knowing it	258 (43.7)	94 (15.9)	239 (40.4)
C4	Heard of vaccination and its protection against HPV infection	166 (28.1)	294 (49.7)	131 (22.2)
C5	HPV vaccine protects against all STIs	264 (44.7)	68 (11.5)	259 (43.8)
C6	The best time to take the HPV vaccine	**Frequency**		
	Before sexual intercourse	207 (35.0)		
	After sexual intercourse	46 (7.8)		
	When one is infected with HPV	123 (20.8)		
	Don’t know	215 (36.4)		

Table 4 presents the findings regarding the assessment of the practice of HPV prevention. Ten questions were asked, and the students responded in a manner that several behaviors can be deduced. 38.2% admitted being in a relationship, of which only 14.9% are in a sexual relationship while 85.1% never had sex. Of those that have had sexual debut, the majority (9.6%) are in the ages between 11 and 13. It was also found that 5.6% had two or more sexual partners, and 10.3% admitted to not using condoms during sexual intercourse. 11.8% have had STDs, and 27.2% had previously undergone HIV screening. Lastly, one out of ten admitted to taking alcohol, 6.3% smoke cigarettes or marijuana, and 6.4% have engaged in substance abuse.

**Table 4:** Assessment of practice of HPV prevention

Code	Variable	Frequency (%)	Code	Variable	Frequency (%)
D1	Have a boyfriend/girlfriend?		D6	Take Alcohol	
	Yes	226 (38.2)		Yes	62 (10.5)
	No	365 (61.8)		No	529 (89.5)
D2	In any sexual relationship?		D7	Smoke cigarettes or marijuana	
	Yes	88 (14.9)		Yes	37 (6.3)
	No	142 (24.0)		No	554 (93.7)
	Not Applicable	361 (61.1)	D8	Engage in substance abuse	
D3	Age (Years) at sexual debut			Yes	38 (6.4)
	Never had sex	503 (85.1)		No	553 (93.6)
	8–10	14 (2.4)	D9	Had any STI/STD?	
	11–13	57 (9.6)		Yes	70 (11.8)
	14–16	8 (1.4)		No	521 (88.2)
	17–19	9 (1.5)	D10	Have done a screening test for HIV/AIDs	
D4	Number of sexual partners been with in the last 6 months			Yes	161 (27.2)
	None	503 (85.1)		No	430 (72.8)
	1	55 (9.3)			
	2 or more	33 (5.6)			
D5	Use condoms during sexual intercourse				
	Never had sex	503 (85.1)			
	Yes	27 (4.6)			
	No	61 (10.3)			

Table 5 presents the findings regarding the assessment of willingness for HPV uptake. There are high levels of agreement regarding willingness to get vaccinated against HPV, perceived willingness of parental consent for vaccination, and willingness to recommend the HPV vaccine to others.

**Table 5:** Assessment of willingness for HPV uptake

Code	Variable	Yes (%)	No (%)
E1	Willing to get vaccinated against HPV	381 (64.5)	210 (35.5)
E2	Parents will be willing to let you take the HPV vaccine	321 (54.3)	270 (45.7)
E3	Will recommend the HPV vaccine to others	374 (63.3)	217 (36.7)

### Bivariate analysis

The data measuring knowledge of HPV infection and sociodemographic variables are nominal, and there was no aggregation of latent variables. Hence, the Mann-Whitney test was used to assess the median differences of knowledge of HPV infection disaggregated by sex and class of study, while the Kruskal-Wallis test was used when the same variables are disaggregated by age, parental occupation, and education. Tribe and religion were excluded because they could be subjected to a different interpretation that ascribes certain sexual behavior or observations to specific tribes or religions. In line with this, Table 6 displays the results of the Mann-Whitney and Kruskal-Wallis tests between the knowledge of HPV infection and sociodemographic variables.

From Table 6, the negative values in sex and class of study mean that males scored lower than females and respondents in JSS scored lower than SSS. All the results can be grouped into three categories. First, those where there are no statistically significant differences in knowledge of HPV infection disaggregated by the sociodemographic variables. Second, those whose *p*-values are less than 0.05 showed moderately statistically significant median differences, while those with *p*-values less than 0.001 showed extremely statistically significant differences. When the *p*-value is not significant, it signifies that the response is purely random, while significance indicates that the knowledge of HPV infection differs clearly across the five sociodemographic variables.

**Table 6:** Median tests of knowledge of HPV infection patterned by the sociodemographic variables

Code	Age	Sex	Parents Occupation	Parents education	Class of study
**B1**	9.01*	-2.92*	9.02	2.98	-4.973**
**B2**	3.81	-1.51	3.44	2.79	-2.39*
**B3**	5.02	-1.5	6.19	3.96	-0.36
**B4**	1.79	-1.13	1.62	4.79	-1.27
**B5**	8.19*	-0.71	7.67	0.67	-2.06*
**B6**	7.63*	-3.16*	12.48*	10.99*	-2.72*
**B7**	10.05*	-1.44	5.94	3.98	-4.07**
**B8**	10.56*	-0.57	6.14	4.03	-4.02**
**B9**	5.36	-1.36	15.21*	8.32*	-4.44**
**C1**	8.94*	-1.5	4.07	2.62	-2.69*
**C2**	4.2	-1.39	14.07*	3.39	-2.13*
**C3**	16.60**	-0.55	10.60*	4.23	-5.21**
**C4**	1.19	-0.68	10.18*	4.34	-3.52**
**C5**	13.76*	-0.35	15.40*	10.14*	-3.97**
**C6**	4.52	-1.92	5.1	3.13	-2.54*
**E1**	14.23*	-0.41	22.65**	20.49**	-4.75**
**E2**	19.94**	-0.77	17.73*	15.02*	-4.73**
**E3**	22.69**	-0.76	26.33**	23.02**	-5.63**

*p value less than 0.05; **p value less than 0.001

The second bivariate analysis involves the use of the Chi-square test of independence to establish the relationship between the practice of HPV prevention and the trio of knowledge of HPV infection, knowledge of HPV primary prevention, and willingness for HPV uptake. The results, as presented in Table 7, can be interpreted in three ways. First, where there are no significant associations, signifying independence. Second, where the *p*-value is less than 0.05, indicating a moderate association at the 5% level of significance. Third, where the *p*-value is less than 0.001, denoting a strong and undeniable significant association.

The results were adequately discussed, and several areas of probable interventions were proposed.

**Table 7:** Summary of the test of association

	D1	D2	D3	D4	D5	D6	D7	D8	D9	D10
**B1**	2.5	3.6	5.53	2.4	4.41	4.59	4.99	5.02	6.21*	1.89
**B2**	1.17	2.33	16.02*	2.56	9.42	5.88	4.73	3.46	2.17	0.89
**B3**	0.84	2.46	7.44	3.82	2.46	1.48	0.4	1.2	0.99	0.88
**B4**	0.06	3.63	10.05	2.64	7.48	6.99*	3.24	2.63	0.71	0.37
**B5**	1.71	2.96	12.14	6.41	1.86	1.78	1.63	1.85	6.49*	0.14
**B6**	1.12	4.94	7.14	1.88	5.05	4.45	2.34	7.22*	15.45**	0.06
**B7**	4.98	6.43	10.48	1.55	4.07	0.85	4.24	4.77	3.92	0.01
**B8**	1.87	2.56	4.7	3.29	5.69	1.18	5.9	17.28**	6.51*	0.11
**B9**	5.81	18.60**	22.04*	20.24**	14.56*	12.31*	29.87**	15.53**	21.48**	1.3
**C1**	0.78	0.97	6.49	3.9	1.47	1.64	2.13	1.47	7.09*	6.07*
**C2**	2.13	3.9	11.7	2.99	5.53	5.23	20.09**	3.44	7.64*	0.31
**C3**	2.37	13.87*	9.97	14.42*	6.62	1.35	2.93	4.9	7.65*	1.24
**C4**	2.24	4.81	6.89	5.2	4.04	27.92**	19.31**	12.17*	16.96**	1.95
C5	1.07	1.25	11.49	2.01	4.34	4.15	4.59	10.68*	14.93*	3.42
C6	8.63*	15.30*	26.21*	16.37*	12.37	5.48	10.80*	19.03**	10.83*	0.47
E1	1.24	1.02	6.68	5.33	6.37*	0.32	1.87	1.5	1.86	25.60**
E2	1.13	1.3	0.94	1.14	0.86	0.52	0.14	0.02	2.37	10.60*
E3	0.03	6.21*	7.8	10.10*	7.98*	0.81	1.45	3.08	6.03*	6.32*

*p value less than 0.05; **p value less than 0.001

Table 8 presents the findings regarding the assessment of willingness for HPV uptake when disaggregated by sex. In all three cases, there are no statistically significant differences between the willingness for HPV uptake and the sex of the young adolescents.

**Table 8:** Relationship between willingness for HPV uptake and sex of the students

Code	Sex	Yes	No	Chi-square
**E1**	Male	137	72	0.166
	Female	244	138	
**E2**	Male	118	91	0.599
	Female	203	179	
**E3**	Male	128	81	0.578
	Female	246	136	

## Discussion

This study assessed the knowledge of HPV infection and practice of primary prevention, the willingness of HPV uptake, and attitudinal differences and correlates among randomly sampled secondary school students across the three senatorial districts of Benue State.

This study found a statistically significant median difference in the perceived knowledge that HPV infection is a sexually transmitted infection (STI) based on age. This is the case of indirectly assessing the general knowledge of STIs. It appears that there are significant knowledge gaps between males and females, among the ages, and between those in junior and senior secondary schools. Generally, younger adolescents tend to have lower awareness compared to older ones. This corroborated a study done in Nigeria [27]. However, the sample in the study was female undergraduate students. Several factors might contribute to this gap. Younger respondents are less likely to be sexually active [28] and, hence, might have less exposure to sexual health education compared to older ones due to age. Additionally, the HPV vaccine’s introduction in recent years might make it a less familiar concept to some younger adolescents who have not encountered it through traditional educational channels in schools, religious centers, or other places. Younger adolescents unaware of HPV’s STI status might engage in risky sexual behavior unintentionally [29]. Since the class of study is based on age, the result follows: students in lower classes have lower HPV and STI knowledge than those in senior secondary. Intervention programs should target young adolescents, particularly at the junior secondary level.

Similarly, females tend to have higher knowledge of HPV compared to men [30]. It may be because many countries do not include males in HPV vaccination and focus on achieving high coverage in females to promote herd protection [31]. More HPV awareness campaigns by countries are directed towards females, especially in cervical cancer awareness, screening, and prevention. As a result, there are disparities in the knowledge level of HPV between males and females.

The result from this study found that there were no statistically significant median differences in the perceived knowledge that HPV can cause penile and cervical cancer based on several demographic factors, including age, gender, parents’ education and occupation, and the level of education of the students. It could indicate that the information about HPV and its link to penile and cervical cancer is generally well-disseminated across different demographics in the surveyed population. Alternatively, it could suggest that there is a lack of awareness or education on this topic across all demographic groups. This result should be interpreted with caution since the samples were comprised of males, who may not have adequate knowledge of cervical cancer, and females, who may not have adequate knowledge of penile cancer. Moreover, penile cancer has been reported to be a rare form of cancer, of which many people may not be aware [32].

The result of statistically significant median differences in the perceived knowledge that HPV can be transmitted by skin-to-skin contact, patterned on various demographic factors, suggests several important points. It indicates that there are differences in knowledge levels across different demographic groups regarding how HPV is transmitted. This could imply that certain groups are not as well-informed about the modes of transmission of HPV, which is crucial for prevention efforts. These differences suggest widespread confusion about how HPV spreads. People might believe HPV is solely transmitted through sexual contact, neglecting the possibility of skin-to-skin transmission [33]. Lack of knowledge about skin-to-skin transmission can lead to underestimating risk factors and potentially increasing the spread of HPV. The misconception that HPV is purely sexually transmitted can lead to a false sense of security, potentially leading to more skin-to-skin transmission [34], especially among younger age groups and those in lower classes. Intervention in this aspect could be routine hygiene in the prevention of nonsexual transmission of HPV.

This study found that age is a significant factor in perceived knowledge of HPV transmission through sexual intercourse, suggesting younger people might have lower awareness compared to older adults. Younger respondents might not have received comprehensive sex education that covers HPV transmission methods. No pattern of knowledge of sexual transmission of HPV based on sex, parental education, or occupation was found. The lack of significant differences based on sex suggests that both males and females in the surveyed population have similar levels of perceived knowledge about HPV transmission via sexual intercourse. The lack of significant differences based on parents’ education and occupation suggests that these factors may not play a significant role in shaping individuals’ perceived knowledge about HPV transmission via sexual intercourse.

This result showed a general high or low level of adequate knowledge of the best time to receive the HPV vaccine. Intervention here will include massive awareness and general sexual and reproductive education. If the level of information is low, then this counters the claims made when the students were asked if they had adequate knowledge of HPV before the main survey. On the other hand, if the level of information is high, it means that the students have received prior information from different sources (government officials, healthcare workers, and school-based vaccination campaigners). The source could be from several public health campaigns in Nigeria that might have effectively provided guidelines on when and how to access the HPV vaccines. The information would have reached different audiences regardless of sociodemographic background.

The result of this study suggests that different age groups may have varying attitudes and beliefs towards HPV vaccination. Younger adolescents may have limited knowledge of the benefits of HPV vaccination and, hence, do not have the necessary knowledge repository to recommend vaccination to their peers.

Secondly, parents might be skeptical about vaccinating younger adolescents, and the respondents are likely to be aware of their parents’ misconceptions about HPV vaccination. This is fueled by the widely held superstitious belief that the HPV vaccine is associated with early sexual initiation and sexual promiscuity [35]. In addition, some of the respondents are from rural areas where vaccine hesitancy is high [36].

Individuals with a past STD may have had more exposure to healthcare services, education, and testing related to STDs, including information about HPV. This increased exposure could have contributed to their greater knowledge about HPV, including the best time to take the HPV vaccine and other routine healthcare services needed for the treatment or management of their previous STD.

People who have had an STD in the past might also be more proactive about seeking information and education on sexual health topics [37], which could further enhance their knowledge about HPV and the importance of vaccination. Their past experience may have made them more receptive to sexual education messages and more likely to engage with healthcare providers regarding preventive measures like the HPV vaccine.

Therefore, past experience with an STD could be a significant factor influencing individuals’ knowledge about HPV and vaccination [38]. Intervention here will be in the form of targeted education and outreach efforts to reach populations at higher risk.

Individuals who had an earlier sexual debut may have had more sexual experiences and therefore may be more aware of the symptoms and consequences of HPV, including genital warts, which corroborates a study done in Iran [39]. This firsthand experience may have led to a greater understanding of the link between HPV and genital warts. Early sexual debut may also be associated with increased exposure to sexual health education or healthcare services, where information about HPV and its consequences, including genital warts, may have been provided. This exposure could contribute to greater knowledge about HPV among those with an earlier sexual debut.

Respondents who take alcohol are likely to know that HPV can cause penile cancer. It could also be that most of the respondents who consume alcohol are male, and as such, they may be in a better position to have knowledge of penile cancer compared to females. This is because penile cancer primarily affects males, so males may be more likely to seek information about this specific type of cancer compared to females. Although alcohol consumption has been linked to penile cancer, it is unlikely that the respondents might have the proper evidence-based knowledge of the alcohol link to penile cancer via HPV infection.

It could be that most males in this study who take alcohol responded differently from the other three groups (males who do not take alcohol and females who do).

The significant association is straightforward: those who had STDs in the past are most likely to know sexual and non-sexual modes of transmission of HPV because of the prior information they had because of their history with STDs. The same applies to oropharyngeal cancer. Technically, only a few of the respondents are aware that HPV can cause oropharyngeal cancer since only a small percentage of the students have either had sex or had STDs in the past.

This research suggests a potential link between non-sexual transmission and engagement in substance abuse. Individuals with lower knowledge about HPV transmission may be more likely to engage in substance abuse, and vice versa. Several factors could explain this association. For instance, individuals who engage in substance abuse may prioritize immediate gratification (like sharing drug syringes) over long-term health concerns, leading to lower awareness about HPV non-sexual transmission. Further research is needed here.

This is another area where further research is needed to understand the interconnectivity between HPV, infertility, and substance abuse. Those struggling with substance abuse might be more receptive to information about health risks in general, leading them to encounter information about HPV and infertility. Research has linked HPV infection as a potential risk factor for female infertility but not as an independent cause [40], but none has directly linked infertility caused by HPV infection to substance abuse.

Individuals who have had any STDs in the past may be more aware of the risks of HPV infection, including infertility, leading to higher knowledge levels. A past STD diagnosis could lead to a greater awareness of sexual health topics in general. This makes them more receptive to information about HPV and its consequences.

Early sexual initiation comes with the risk of unwanted pregnancies and STDs. Thankfully, research has shown that HIV testing is a strong predictor of HPV vaccination uptake [41]. Early sexual debuts can indeed be associated with higher odds of seeking HPV/HIV screening. Early initiation of sexual activity is linked to increased exposure to sexually transmitted infections (STIs), including HPV and HIV. Individuals who start having sex at a younger age may be more aware of their potential risks and therefore more likely to seek screening services to protect their health. The recommended intervention is early and comprehensive sexual education, as well as access to screening services, to prevent and detect STIs.

Being in a sexual relationship has been found in this study to be associated with having HPV for many years without knowing it. This is unsurprising, as sexual intercourse or being in a sexual relationship increases the likelihood of HPV infection [42], which often has no signs or symptoms. People can be infected with HPV for years without knowing it because the virus can remain dormant in the body for long periods of time [43]. In many cases, the immune system clears the virus naturally within a couple of years. However, in some cases, HPV can persist and lead to health problems such as genital warts or HPV-related cancers. Being in a sexual relationship increases the likelihood of sexual transmission of HPV.

This study found that the best time to take the HPV vaccine is associated with the age (years) at sexual debut. The HPV vaccine is most effective when given before exposure to the virus through sexual activity or via skin-to-skin transmission. Therefore, the recommended age for HPV vaccination is before the onset of sexual activity [44]. This result could be explained in two ways: those who have yet to have a sexual debut do not know the best time to take the HPV vaccine, or those who have had sex know the best time to take HPV.

Intervention here could involve using evidence-based approaches to educate those who have not had sex on the need to take the HPV vaccine. This could be combined with campaigns to dispel any misconceptions or myths around the HPV vaccine, sexual promiscuity, indiscriminate sexual behavior, and sexual experimentation.

It appears to be a straight-line equation. The findings from this study showed a significant association between condom use and willingness to get vaccinated against HPV infection. The interpretation could be that the respondents who use condoms during sexual intercourse are more willing to take other preventive measures, such as getting vaccinated against HPV. This may be due to heightened consciousness, which makes them more receptive to any recommended preventive measures, like HPV vaccination. Technically, those who perceive themselves as at a higher risk of STIs due to their sexual behavior might be more motivated to get vaccinated against HPV as an additional layer of protection [45]. However, it should be noted that the decision to get vaccinated against HPV is influenced by many factors beyond just condom use. Some of the factors are listed for different populations: migrants and refugees [46], United States rural populations [47], English Canada [48], and Sub-Saharan Africa [49], amongst others.

The association between willingness to get vaccinated against HPV and having ever done HIV/AIDS screening might suggest that adolescents who have done HIV/AIDS screening are more aware of preventive health measures and more likely to be willing to get vaccinated against HPV. This can make them more receptive to other preventive measures, like the use of condoms and HIV/AIDS testing. Adolescents who are proactive about their sexual health might be more likely to seek out both HPV vaccination and HIV/AIDS testing.

People who consistently use condoms might be more health-conscious in general, making them proactive and more likely to understand the value of preventive measures like HPV vaccination and recommend them to others. Those who perceive themselves as at a higher risk of STIs due to their sexual behavior, especially with multiple partners, might be more motivated to recommend HPV vaccination as an additional prevention layer, even if they themselves use condoms [50]. Also, several factors not mentioned in this study can contribute to a positive association between condom use during sexual intercourse and the willingness to get vaccinated against HPV, as both behaviors are indicative of a proactive approach to sexual health.

The findings that there are no statistically significant differences between the sex of young adolescents and their willingness to get vaccinated against HPV, their parents’ willingness to let them take the HPV vaccine, and their likelihood to recommend the HPV vaccine to others have important implications for ongoing HPV mass vaccination efforts in Nigeria. These results suggest that sex may not be a determining factor in the acceptance and promotion of the HPV vaccine among young adolescents and their parents. However, this disagreed with earlier findings that parents’ willingness is based on the sex of the adolescent [51–53]. Sadly, only girls are currently being vaccinated across the country. If males are included in the vaccination, the implications of the study are as follows:

- The vaccination campaigns can use uniform messages and strategies for both boys and girls. This simplifies the design and implementation of educational materials and outreach programs and hence reduces the cost involved in tailoring messages to specific sex. Hence, increasing overall vaccination rates without needing to tailor messages separately for boys and girls.
- With sex not being a significant differentiator as this study has shown, it is therefore important to focus on other underlying factors that may influence vaccine acceptance, such as socio-economic status, educational background, cultural beliefs, and accessibility of vaccination services. Understanding the barriers will help in designing interventions to address them.
- Since parental willingness to vaccinate their children does not significantly differ by the sex of the adolescent as this study has found, deliberate efforts should be made to educate parents uniformly. Providing comprehensive evidence-based information on the benefits and safety of the HPV vaccine can encourage parental consent across the board.
- The finding from this study that both boys and girls are equally likely to recommend the HPV vaccine to others suggests that adolescents can be effective advocates for the vaccine within their peer groups. Schools, NGOs, and community programs can engage trained adolescents as ambassadors for HPV vaccination, leveraging their ability to influence their peers regardless of gender.
- Finally, this paper strongly advocates for the inclusion of adolescent boys into the ongoing HPV mass vaccinations across the developing countries. Hence by ensuring that both boys and girls are equally likely to be vaccinated, long-term health benefits such as reduced incidence of HPV-related cancers (e.g., cervical cancer in women and throat cancer in men) can be more uniformly realized across the population [54].

### Limitation

The study acknowledged some limitations. First, a recall bias might occur because a greater percentage of questions involved recalling past events in their lives. Second, the study’s reliance on self-reported data may introduce response bias, as participants may provide answers they perceive as socially desirable rather than reflecting their true knowledge or behaviors. This is because the interviewers were trained not to interfere, as only minimal questions were clarified. Finally, information regarding caregivers’ willingness to consent relies on participants’ responses, making it subjective. Ideally, data directly from caregivers would provide a more objective assessment.

### Policy recommendations

Ten actionable recommendations are prescribed and presented in Table 9.

**Table 9:** Actionable Recommendations for Policymakers

Theme	Action	Recommendation
Age disparities in HPV knowledge	Develop targeted educational programs for younger adolescents	Implement comprehensive sex education curriculums in junior secondary schools (JSS) to increase HPV awareness and knowledge among younger students
Gender disparities in HPV knowledge	Increase HPV awareness campaigns focused on males	Design and launch HPV awareness campaigns that specifically target males, highlighting the importance of HPV vaccination for both genders and addressing misconceptions about HPV being solely a female concern
Knowledge gaps in HPV and cancer link	Provide evidence-based information on HPV-related cancers in educational materials	Ensure that HPV education includes information on the link between HPV and both penile and cervical cancers, targeting both male and female students
Non-sexual transmission awareness	Include information on non-sexual transmission of HPV in health education programs	Educate adolescents about the various modes of HPV transmission, including skin-to-skin contact, to reduce misconceptions and promote comprehensive prevention strategies
Vaccination timing knowledge	Promote awareness of the best time to receive the HPV vaccine	Conduct campaigns to educate young adolescents and their caregivers about the WHO recommended age for HPV vaccination, emphasizing the importance of getting vaccinated before the onset of sexual activity
Parental influence on vaccination	Address parental misconceptions and promote vaccine acceptance	Develop outreach programs aimed at educating parents about the safety and benefits of the HPV vaccine, dispelling myths related to early sexual initiation and promiscuity
Health-seeking behaviors	Campaign for routine health check-ups and screenings for adolescents	Integrate HPV education and vaccination promotion into routine health check-ups (routinization) and sexual health services for adolescents, leveraging existing primary healthcare infrastructure to increase vaccine uptake
Condom use and HPV awareness	Promote condom use as part of a broader sexual health education program	Enhance sexual health education programs especially in schools to include information about the benefits of condom use and its role in preventing HPV transmission, along with promoting HPV vaccination as an additional layer of protection
Equity in health education	Ensure that are strategies for HPV education programs to reach all socioeconomic and geographical groups	Create HPV education and vaccination programs specifically designed for underserved communities, including those in low-income areas, rural locations, and facing social barriers or having physical and developmental disabilities. Making sure everyone has a fair chance to learn about HPV and get vaccinated
Support for underserved populations	Provide additional resources and support to underserved populations for HPV vaccination	Collaborate with community and religious leaders, influencers and organizations to offer free or subsidized HPV vaccinations, transportation, and culturally sensitive educational materials to underserved populations to increase vaccine uptake and equity

## Conclusion

The study highlighted several significant findings regarding the perceived knowledge and behaviors related to HPV infection and vaccination. It revealed age disparities in awareness, with younger individuals generally less informed about HPV as a sexually transmitted infection (STI) compared to their older counterparts. Females exhibited greater knowledge of HPV, possibly influenced by targeted awareness campaigns focused on cervical cancer prevention. Interestingly, there was no significant difference in knowledge regarding HPV’s link to cancer based on demographic factors, suggesting widespread dissemination of this information. However, gaps exist in understanding non-sexual transmission and the best time for vaccination, emphasizing the need for targeted intervention in the form of sexual and reproductive education. Additionally, there was a significant association between past STDs and knowledge about HPV, indicating a potential link between health-seeking behaviors and HPV awareness. Public health campaigns can leverage existing infrastructure, and healthcare providers can play a key role in raising HPV awareness. Additionally, positive associations between condom use, past STDs, and HIV testing and HPV awareness suggest opportunities for integrated interventions to promote HPV vaccination and safer sexual practices among young adolescents.

## Data Availability

The data that support the findings of this study are available from the corresponding author upon reasonable request.

## Abbreviations

HIV: Human Immunodeficiency Virus
HPV: Human Papillomavirus
JSS: Junior Secondary School
LGA: Local Government Area
SSS: Senior Secondary School
STD: Sexually Transmitted Disease
STI: Sexually Transmitted Infection
